# Population dynamics and spatial structure of the grey rockcod (*Lepidonotothen squamifrons*) in the vicinity of Heard Island and the McDonald Islands

**DOI:** 10.1371/journal.pone.0298754

**Published:** 2024-05-14

**Authors:** Dale Maschette, Paul Burch, Bryn Farmer, Emma Woodcock, Clara Péron, Breanna Cramer, Caleb Gardner, Dirk C. Welsford

**Affiliations:** 1 Department of Climate Change, Australian Antarctic Division, Energy, The Environment and Water, Kingston, Tasmania, Australia; 2 Institute for Marine and Antarctic Studies, Fisheries and Aquaculture Centre, University of Tasmania, Hobart, Tasmania, Australia; 3 CSIRO Oceans and Atmosphere, Hobart, Tasmania, Australia; 4 Laboratoire de Biologie des Organismes et Ecosystèmes Aquatiques MNHN, Paris, France; 5 APC Prosthetics Northmead NSW, Northmead, NSW, Australia; MARE – Marine and Environmental Sciences Centre, PORTUGAL

## Abstract

The grey rockcod, *Lepidonotothen squamifrons* is an important prey species for seals, penguins and Patagonian toothfish (*Dissostichus eleginoides*) in the Southern Ocean. Across the Kerguelen Plateau, the species was fished to commercial extinction (*ca*. 152 000 tonnes between 1971 and 1978) prior to the declaration of the French Exclusive Economic Zone in 1979 and the Australian Fishing Zone in 1981. In this study we estimate; age, growth, maturity, sex ratio, body condition (weight-at-length), and population density of grey rockcod using data from 19 trawl surveys from 1990 to 2014. There appeared to be three distinct geographical populations, with differences in biological parameters within each population. This study has identified separate metapopulations within the southern region of the Kerguelen Plateau and we recommend that management should take into account the different characteristics of these populations, and that this meta-population structure may be a factor in why this species required several decades to show signs of recovery.

## Introduction

Hutchings [1] stated that exploitation affects life history. Despite this, there are a myriad of potential reasons explaining why an exploited population of fish either never, or takes a prolonged period to recover [2]. On a broad scale, the mass removal of a population can potentially lead to population densities too low to allow for population recovery and the distortion of the associated food web. Within an exploited population, effects can be phenotypic and/or genetic changes [1,3]. These changes can be represented in a number of ways, such as changes in size/age of maturity, weight/length at age or, levels of fecundity [4]. A number of species across the world have exhibited at least some, if not all, of these changes after exploitation, including haddock (*Melanogrammus aeglefinus*), Pacific salmon (*Oncorhynchus* spp.) and northern cod (*Gadus morhua*) [1,3,4]. However, despite a similar history of overexploitation, species in high latitudes of the southern hemisphere have received less scrutiny (although see works by Kock [5–7] and Constable [8]).

Among the first fish species to be exploited on the Kerguelen Plateau was the grey rockcod (*Lepidonotothen squamifrons*), first described by Günther [9]. It is a demersal fish species, with a planktonic post hatching early life stage, endemic to the Southern Ocean and predominantly found in depths <570m, although some specimens have been caught in waters as deep as 700m in the vicinity of South Georgia and Shag Rocks and 950m within the Heard Island and McDonald Islands (HIMI) area [10–13]. This species was among the first of the fish species to be exploited on the Kerguelen Plateau, a large submarine plateau located in the Indian sector of the Southern Ocean [7,14]. Commercial fishing by vessels from the Soviet Union was so intense during the 1970s, with around 152 000 tonnes taken between 1971 and 1978, that the stock approached commercial extinction [15]. The declaration of the French Exclusive Economic Zone (EEZ) and Australian Fishing Zone in 1979 and 1981, respectively, and the establishment of the Commission for the Conservation of Antarctic Marine Living Resources (CCAMLR) in 1982, led to the end of this fishery.

Studies conducted in the Northern Kerguelen Plateau (within the French EEZ) and South Georgia indicate that grey rockcod plays an important role both as a predator of zooplankton, targeting primarily salps, cnidarians and ctenarians, and as prey, being targeted by a wide range of predators including mammals, birds, fish and sharks [11,14,16–22]. This important role in the ecosystem as both predator and prey highlights the need to gain a better understanding of its lifecycle and biology, the food web implications of massive historical removals, and the species potential for recovery.

There are key pieces of information that are required in order to adequately manage a species, these include but are not limited to, population structure, age structure, growth parameters, mortality (both natural and fishing), length-weight relationships and reproduction biology data.

Previous studies have shown differences in biology (such as growth and spawning times) across geographical populations within the grey rockcod range [11,23,24], however they were limited to 2 geographic areas, namely the northern half of the Kerguelen Plateau and Crozet Island; and South Georgia Island (including Shag Rocks).

Constable et al., [25] investigated the status of the grey rockcod stock within the HIMI area, however they treated the entire area as one population for the purpose of their assessment. Additionally, the work by Constable et al., [25], whilst estimating population size, largely used biological parameters from those derived in the Kerguelen plateau to the north. The availability of bycatch data obtained from the Patagonian toothfish fishery operating in the region since 1997 and data from the research surveys conducted in 1990, 1992, 1993, and from 1997 through to 2014 [15], provided new insights to compare to Constable et al., [25].

The aim of this study is to fill some of the key gaps surrounding the biological parameters of grey rockcod in the Southern Kerguelen Plateau within the HIMI EEZ, including age estimation using archived historical samples of otoliths, to allow for future assessments of its population trajectory post over-exploitation. We explored the potential effect of geographical locations (‘grounds’) on the (sub/meta)-population parameters such as growth, maturity, sex ratio, population density and weight at length relationships and their possible implications for stock recovery at HIMI. Finally, we propose a potential source-sink connectivity driven by transport of larvae by ocean currents.

## Methods

### Sample collection

Three research surveys were conducted using bottom trawl within the Heard Island and McDonald Islands EEZ between 1990 and 1993 prior to the commencement of Australia’s commercial fishing in the region (S1 Table). Since the outset of commercial fishing, 19 Random Stratified Trawl Surveys (RSTS) have been conducted each year between April and July (1997–2014). It is worth noting that in 2010, a second RSTS survey was completed later in the year (September) due to mechanical issues during the first, however the grey rockcod sampling was not affected by this issue and both surveys were used (S1 Table). All surveys were conducted with the permission and under the direction of the Australian Antarctic Division and Parks Australia. Typically, research surveys contained 66–79 survey stations, and RSTS contain typically more than 130 stations (see S1 Table). Surveys covered waters down to a depth of 1000m, including those inside the HIMI Marine Reserve [15,26,27]. Each year, fishing stations were randomly chosen with a minimum of 5 nm between stations within 13 strata. These strata are research areas defined according to their ecological relevance and/or their level of commercial fishing effort. At each station, fishing location (using GPS), haul start and finish time were recorded [27,28]. Hauls were conducted using a ‘Champion’ net with a 152 mm mesh, a 50 mm cod end liner and a net opening of 19 m [27]. Tow duration was 30 minutes once the net reached the bottom at a speed of 3 knots. When possible the weight was recorded for all finfish to the lowest taxonomic level, generally species or genus [27,28]. A proportion of individuals from the catch were also measured (for total and standard length), weighed, sexed and analysed for maturity stage (e.g Nowara et al. [27]). In addition to the above, during early surveys (1990–1993) and the 2014 survey sagittal otolith pairs from individuals of a number of species including the grey rockcod were collected to enable age estimation of specimens. Data and specimens collected from these surveys, including otoliths used in this study, were accessed from archives at the Australian Antarctic Division (AAD) [13]. As samples were collected during commercial fishing operations ethics were not required under Australian guidelines.

### Geographical distributions

**Fig 1:**
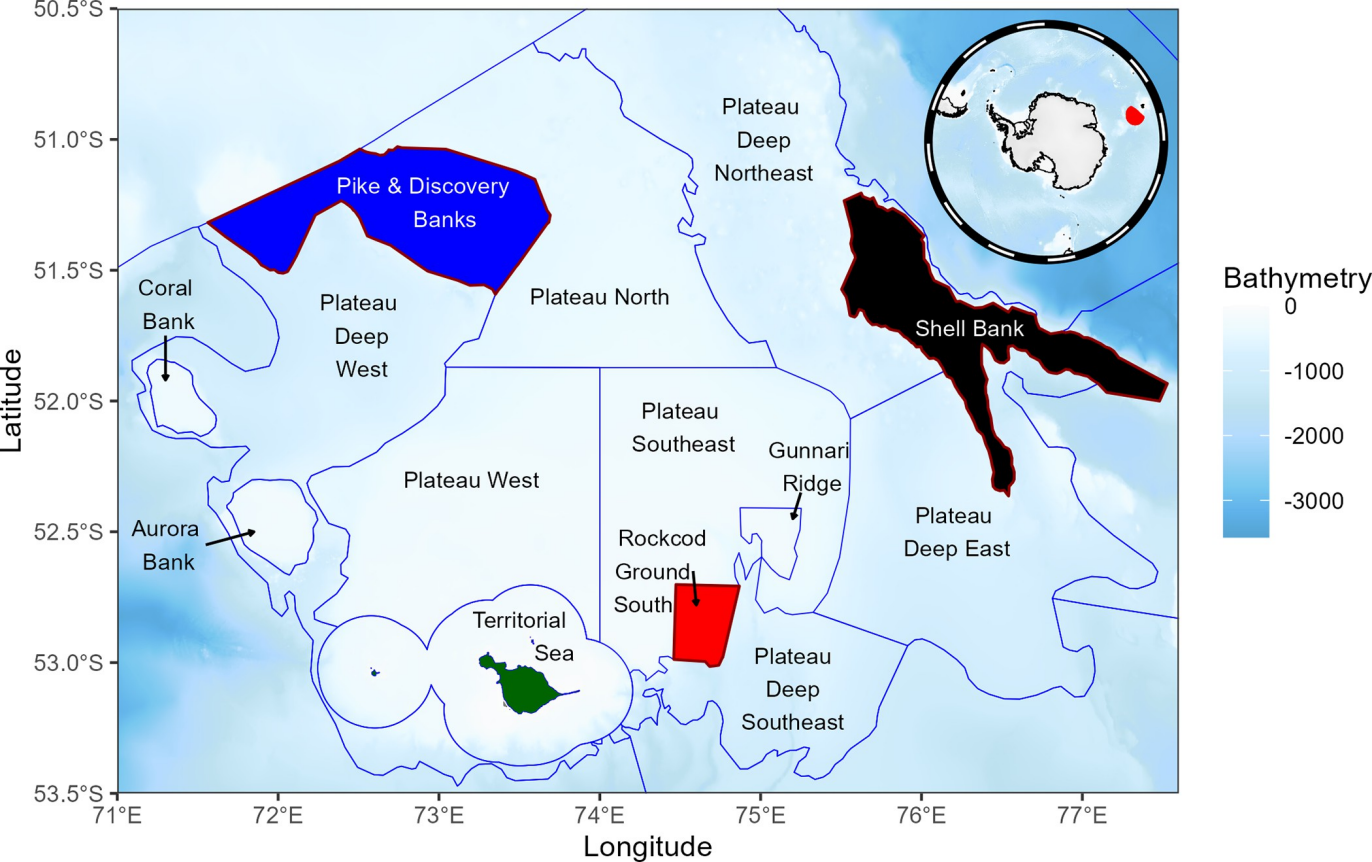
Research strata within the vicinity of Heard Island and McDonald Islands strata covering the distribution of grey rockcod are Rockcod Grounds South, Pike & Discovery Banks and Shell Bank, blue lines indicate strata boundaries, bathymetry data obtained from Geoscience Australia [30].

The distribution and abundance of grey rockcod across the plateau were mapped and aggregated (averaged) on a 0.1° x 0.1° grid using catch locations from research surveys conducted between 1990 and 2014. Mapping was conducted using the *raster* package in R [29] with high resolution bathymetry data (0.001-arcdegree) obtained from Geoscience Australia [30]. The HIMI EEZ is currently separated into different areas or ‘strata’ and were defined according to their habitat characteristics and/or their level of commercial fishing effort respectively [28,31]. There are three strata which covers the main geographical extent of grey rockcod; Rockcod Ground South, Pike & Discovery Banks and Shell Bank (Fig 1).

### Body condition index—length weight relationship

The relationship of length to weight was used as a proxy for fish body condition [32]. The length to weight relationship was fitted by the method of non-linear regression using the *nls* function [33] in R [34], with:

$$W=aL^{b}$$

where *W* is the weight in g, *L* is the total length in mm, *a* relates to the density of the fish and *b* relates to its three dimensional shape.

This relationship was fitted to measurements taken within each survey year and for all years combined (see S2 Table).

In order to visualise and compare what the biological effects of changes in length- weight parameters actually have, comparisons of the weight of grey rockcod at a length of 300 and 400 mm were made between individual years and all years combined across locations and compared using pairwise ANOVA.

### Length frequency and density

Length frequency data was collated for the three strata where grey rockcod occur for applicable survey years. Each year a proportion of individuals from the catch was measured for total and standard length. Due to the small number of rockcod measured each year length frequency data were aggregated over surveys to display overall differences.

Density of fish *D*_*l*_ at length *l* in fish (numbers) per km^2^ was estimated for each haul as:

$$D_{l}=\frac{N_{l}W}{Aw}$$

where *N*_*l*_ is the numbers at length *l*, *A* is the swept area of net for that haul calculated as width of the net (19 m) multiplied by the tow distance in km^2^, *W* is the total catch weight of the haul, and *w* is the total weight of the sample from the haul. Total fish numbers by haul were then averaged across all hauls in a survey stratum to obtain density within each strata. Four years were excluded (1993, 1997, 1998 and 2009) due to insufficient sample sizes within the three strata areas.

Manova was used to explore for differences between the three strata in annual mean fish density and calculated fish weight at 400 mm total length. Where differences were identified a Tukey honest significant differences (TukeyHSD) test was conducted for each response to determine which differences between strata were significant.

### Maturity and sex ratio

The analysis of gonads allows separation of immature and mature fish [35]. Sex and gonad maturation stage were determined macroscopically following the Kock and Kellerman scale [36] where 1 is immature and 5 is spent. Mature fish were defined as those with gonads at stage 2 (developing / resting) or more. Length (Total) at 50% sexual maturity was estimated for each sex using binomial generalised linear models (GLM) in R [34]. Nested models were compared separately using pairwise ANOVA for differences between locations and between each sex within each location.

Sex ratios at each geographical location were calculated and compared using binomial GLM with pairwise comparison.

### Age estimation

A total of 1010 otoliths, ranging in size from *ca*. 3–9 mm, were obtained from the Australian Antarctic Divisions otolith collection [13]. Since the grey rockcod otoliths examined in the present study were collected historically and that fishing in the Southern Ocean does not occur all year round, otolith samples that are suitable to conduct typical age validation methods such as marginal increment analysis do not exist [37–39]. Inspection of the whole and sectioned sagittal otoliths of grey rockcod revealed alternating opaque and translucent layers around a central nucleus (S1 Fig).

Due to the relatively small size of the otoliths (<10 mm in the longest axis) and the shallow sulcal groove, the primordium on each otolith was marked prior to block mounting. Otolith processing was an updated method from that outlined by Welsford et al. [40] developed for Patagonian toothfish (*Dissostichus eleginoides*; confamilial with *L*. *squamifrons*). Briefly, otoliths were mounted within epoxy resin blocks and three sections *ca*. 300–350 μm were cut (positioned so as one section would contain the primordium) using an Isomet low speed saw. Sections were in turn mounted on slides and photographed using a transmitted light, through a microscope at 3.6x – 6x magnification. Otolith quality and clarity was ranked from 1–5 based upon similar criteria used by Lewis and Mackie [41] with 5 being excellent quality and 1 being unreadable.

Reference collections have become a standard method of quality control both temporally for an individual reader and between separate readers [42,43]. A reference collection was compiled of 200 otoliths randomly selected from the collection. The reference collection was aged by the primary reader and an experienced reader from the Australian Antarctic Division in order to investigate consistency and accuracy. Reader age determinations were then compared using a two sample Kolmogorov-Smirnov test at an alpha level of 5% as well as, the index of average percent error (IAPE) and the coefficient of variation (CV) [44]. A minimum of two weeks was maintained between repeat reads of an otolith to ensure that the primary reader had no familiarity with the otolith.

All ageing was carried out by the primary reader, with each specimen read twice without reference to previous age estimates. A nominal birthdate of 1 December was assumed for the age estimation, this was based on an October/November spawning and to coincide with the start of the CCAMLR season.

### Growth curves and subpopulation comparisons

A von Bertalanffy growth model [45] was used to estimate length *L* at age *t* following:

$$L_{\left(t\right)}=L_{\infty }(1-e^{-k(t-t_{0})})$$

Where *L*_*∞*_ is the hypothetical length at age *∞*, *k* is a constant related to the growth rate and *t*_*0*_ the hypothetical age at length zero. The growth model was fitted by the method of non-linear regression using the *nls* function [33] in R [34].

The model was generalised to allow parameters to vary among two or more groups, and the performance of alternative models was evaluated using the Akaike Information Criterion (AIC, [46]), with the model having the lowest AIC being considered optimal and any models within 2 units were considered to be plausible. In this study, models were compared to explore differences between growth parameters for each stratum. Comparisons were conducted using AIC on models containing: parameters for growth of each stratum; fitting either a separate *k*, *t*_*0*_ or *L*_*∞*_ parameter; fitting two separate parameters (retaining only a common *k* or *t*_*0*_); or fitting all parameters separate for each stratum. Due to a lack of age information available for fish from Rockcod Ground South, models were only constructed for Pike & Discovery Banks and Shell Bank.

## Results

### Geographical distributions

**Fig 2:**
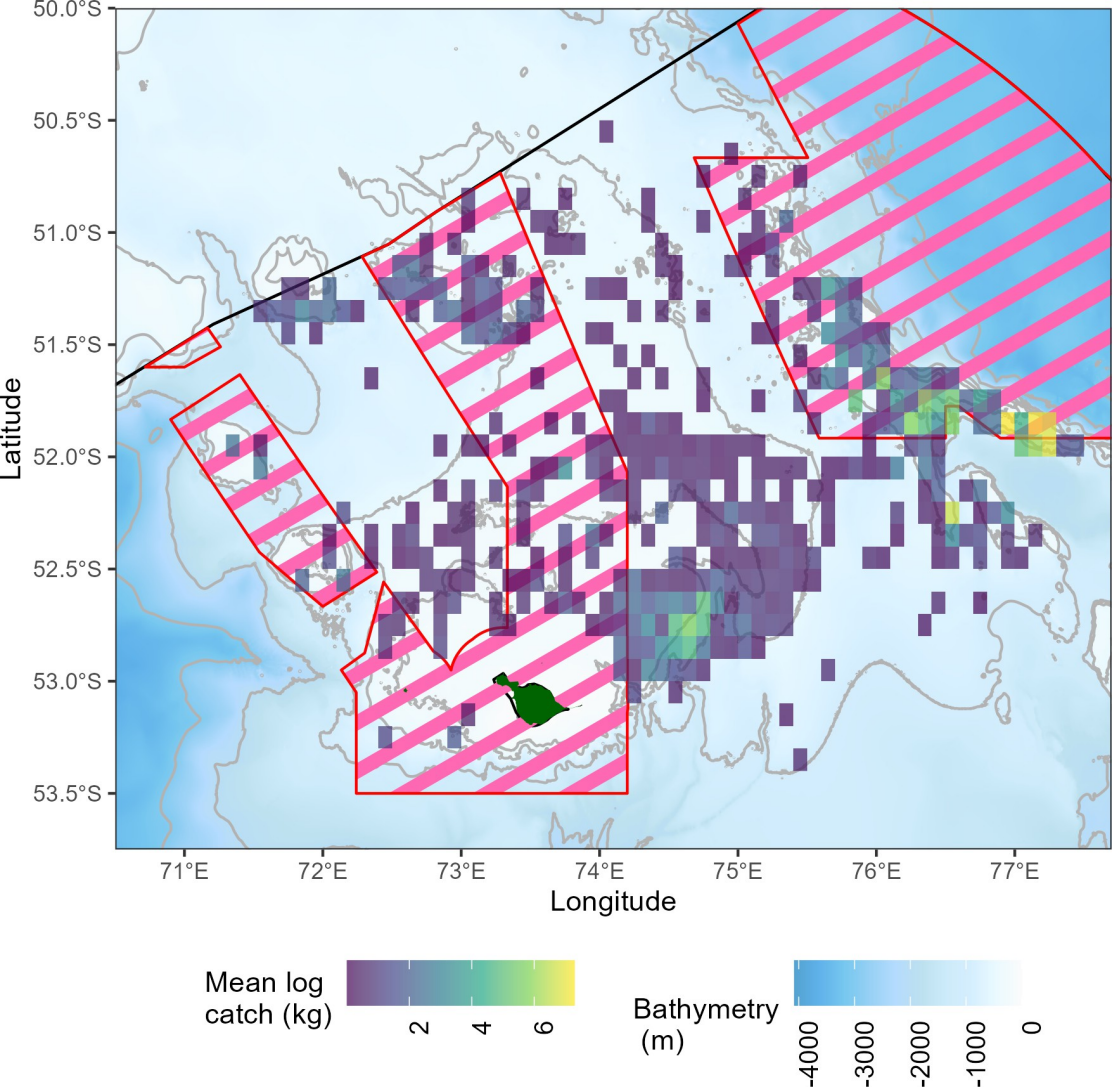
Log mean catch (kg) of grey rockcod (*Lepidonotothen squamifrons*) caught within research surveys (1990–2014) within the vicinity of Heard Island and McDonald Islands (green) plotted at a 0.1° x 0.1° resolution. Pink lines indicate marine protected areas as defined in 2014 [39], bathymetry data obtained from Geoscience Australia [30].

Catches of grey rockcod during research surveys were mapped and aggregated (averaged) on a 0.1° x 0.1° grid. Fig 2 showed consistent clustering of high catches across years at three strata: Shell Bank, Rockcod Ground South and Pike & Discovery Banks. High catches of grey rockcod were also observed at Coral and Aurora Banks, whereas catches/fish density was low in other sampled areas.

### Body condition index—length-weight relationship

**Fig 3:**
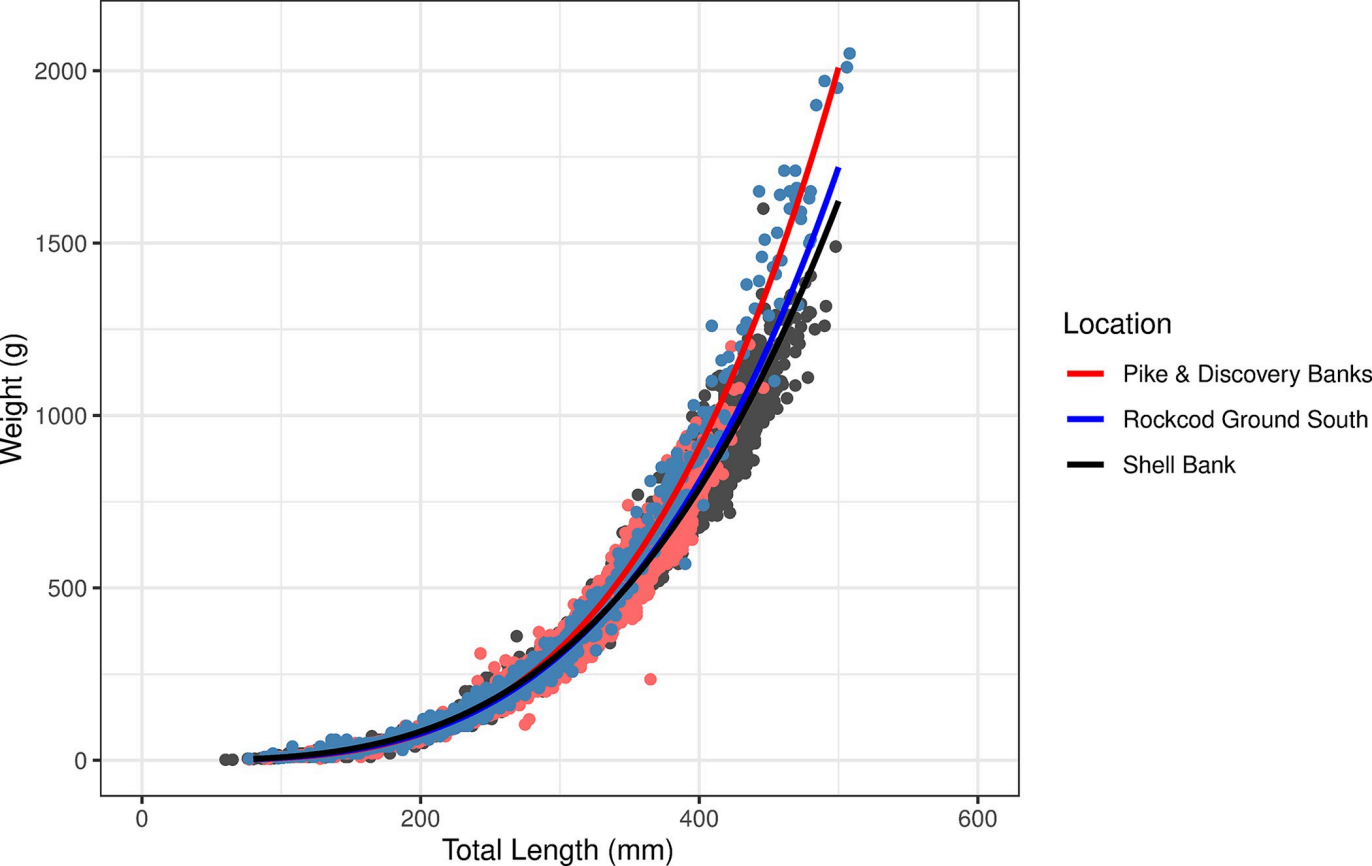
Length weight relationship for grey rockcod (*Lepidonotothen squamifrons*) across three locations within the vicinity of Heard Island and McDonald Islands; Rockcod Ground South (red), Shell Bank (black) and Pike & Discovery Banks (blue) 1990–2014.

The length-weight relationships varied between strata and years (S2 Table). The combined years length-weight relationships showed similar relationships between Rockcod Ground South and Shell Bank whilst the relationship at Pike and Discovery Banks indicated fish were heavier than at other locations (Table 1, Fig 3).

**Table 1:** Ranges of combined years body length and weight and estimates of parameters a and b of the length-weight relationship of *Lepidonotothen squamifrons* using data collected from three locations within the vicinity of Heard Island and McDonald Islands during research surveys.

Location	Number	Minimum Length (mm)	Maximum Length (mm)	Minimum Weight (mm)	Maximum Weight (g)	a	b
Shell Bank	7113	60	550	2	1600	5.29E-07	3.55
Pike & Discovery Banks	1251	77	508	5	2050	2.75E-06	3.25
Rockcod Ground South	5765	77	600	3	2240	1.80E-06	3.32

**Fig 4:**
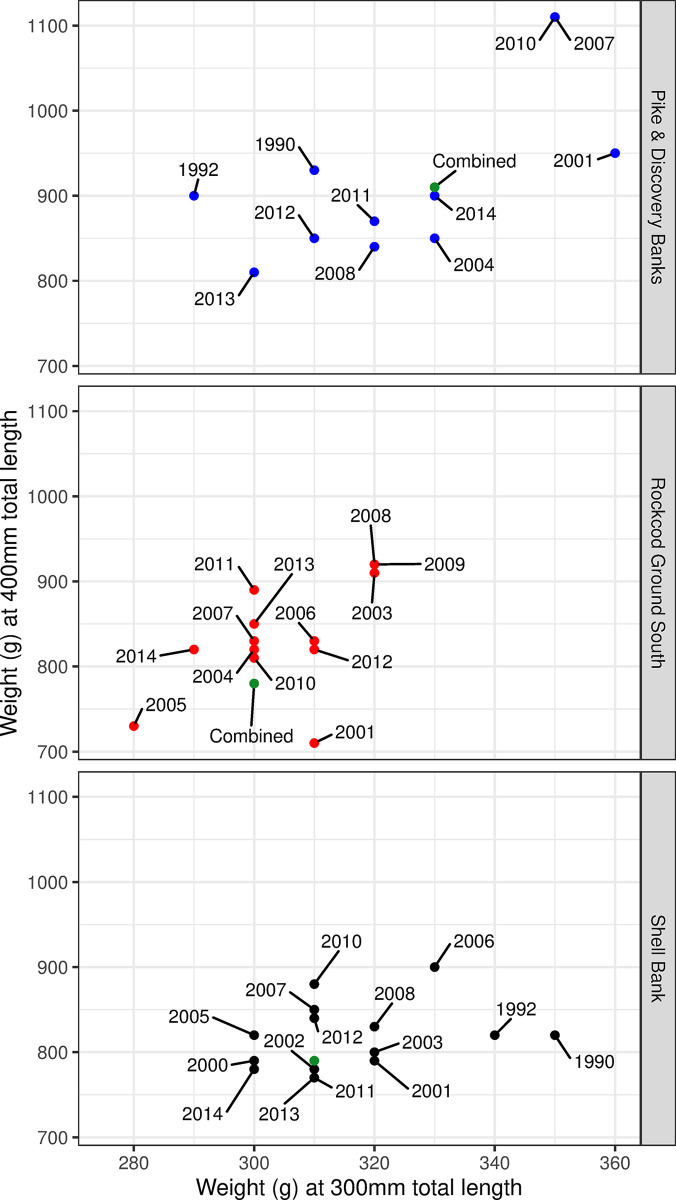
Comparison of estimated weight at 300 mm and 400 mm for grey rockcod (*Lepidonotothen squamifrons*) through various years and all years combined across three locations within the vicinity of Heard Island and McDonald Islands; Rockcod Ground South (A), Pike & Discovery Banks (B) and Shell Bank (C).

Comparisons of the weight of grey rockcod at a length of 300 and 400 mm made between individual years and all years combined indicated a varying range of weights for fish of 300 mm (280–320, 290–360, 30–350 g for Rockcod Ground South, Pike & Discovery Banks and Shell Bank respectively) and 400 mm (710–920, 810–1110, 770–900 g for Rockcod Ground South, Pike & Discovery Banks and Shell Bank respectively) across years at all locations (Fig 4A–4C). ANOVA between locations for both 300 mm and 400 mm grey rockcod showed that fish at Pike & Discovery Banks were significantly heavier compared to those at Shell Bank and Rockcod Ground South (*p* = 0.02 and <0.01, respectively).

### Length frequency and density

**Fig 5:**
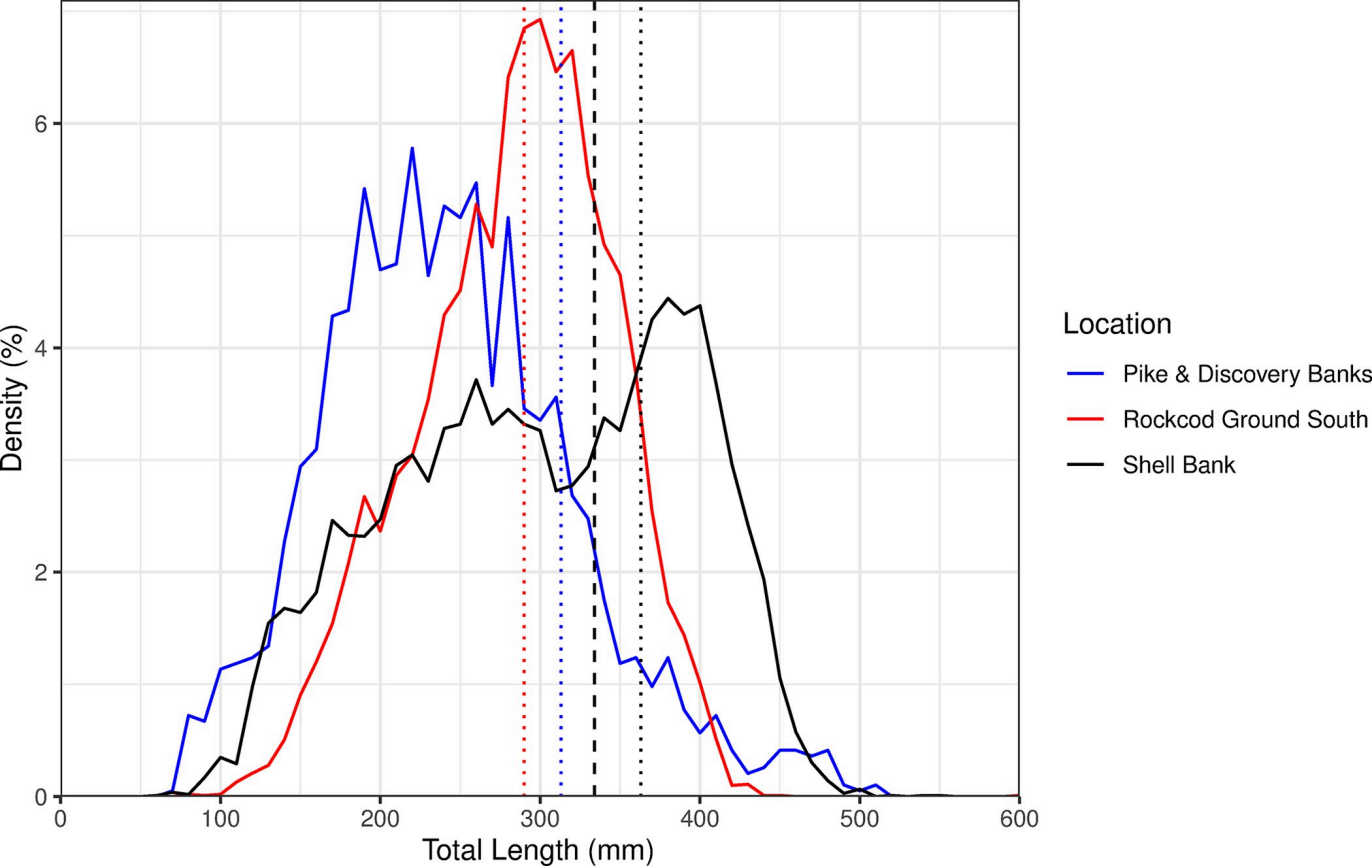
**Length density distribution for grey rockcod (*Lepidonotothen squamifrons*) in the vicinity of Heard Island and McDonald Islands, and all years (1990–2014); Rockcod Ground South (red), Shell Bank (black) and Pike & Discovery Banks (blue).** Dotted lines indicate size of 50% sexual maturity for both sexes in Rockcod Ground South (red) and Pike & Discovery Banks (blue), 50% sexual maturity in Shell Bank (black) is shown for females (dashed) and males (dotted)(see Fig 7).

The largest fish in all three regions, which was caught in 2001, was at Rockcod Ground South and had a total length of 600 mm, with the next largest fish in this area being 446 mm. The largest fish in Pike & Discovery Banks and Shell Bank were 508 mm and 550 mm respectively (Table 1). Length frequency distributions were unimodal within Rockcod Ground South and Pike & Discovery Banks centring at approximately 300 mm and 240 mm, respectively. In contrast, Shell Bank showed a bimodal distribution with a smaller mode at around 250 mm and a larger mode at 400 mm (Fig 5).

**Fig 6:**
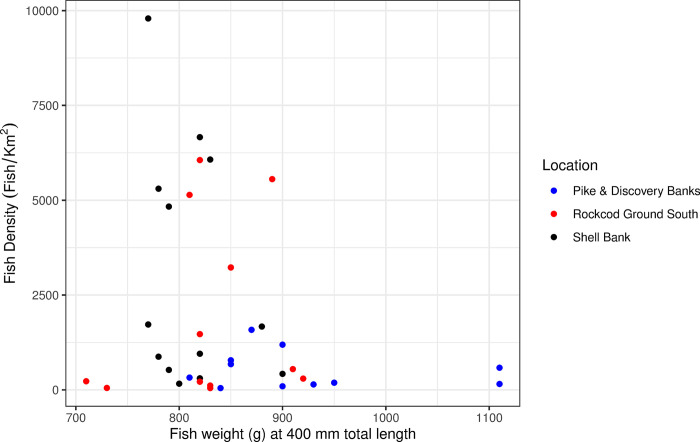
Comparison of annual fish density with weight at a length of 400 mm within the vicinity of Heard Island and McDonald Islands; Pike & Discovery Banks (blue), Shell Bank (black) and Rockcod Ground South (red) for grey rockcod (*Lepidonotothen squamifrons*).

The densities of fish, n/km^2^ was calculated for Rockcod Ground South, Shell Bank and Pike & Discovery Banks (Table 2). Densities showed variation across years both within and across locations. This is potentially due to low sample sizes within each location in some years combined with the occasional aggregating nature of this species. When exploring for differences between the three strata in annual mean fish density and calculated fish weight at 400 mm total length (Fig 6) manova indicated significant evidence for differences between the three locations (*p* = 0.005, DF = 4, Deviance = 4.14). Between the two response variables weight at 400mm total length was more significant (*p* = 0.002, DF = 2, Deviance = 7.74) than fish density (*p* = 0.047, DF = 2, Deviance = 3.34) TukeyHSD test for fish weight at 400mm indicated that the largest difference were between Pike & Discovery Banks with Rockcod Ground South (*adjusted p* = 0.009) and Pike & Discovery Banks with Shell bank (*adjusted p* = 0.002). Negligible difference was estimated between Shell Bank and Rockcod Ground South (*adjusted p* = 0.892). TukeyHSD test for density indicated a large difference between Shell Bank and Pike & Discovery Banks (*adjusted p* = 0.037), whilst limited difference was seen between Shell Bank and Rockcod Ground South (*adjusted p* = 0.475) and between Rockcod Ground South with Pike & Discovery Banks (*adjusted p* = 0.347).

**Table 2:** Density estimates (fish n/km^2^) of grey rockcod (*Lepidonotothen squamifrons*) from three locations within the vicinity of Heard Island and McDonald Islands. Standard errors are shown in parentheses.

Year:	Shell Bank	Pike & Discovery Banks	Rockcod Ground South
1990	952 (±4.74)	143 (±1.49)	-
1992	6662 (±36.81)	94 (±1.63)	-
1999	13112 (±144.77)	265 (±2.25)	-
2000	4832 (±13.45)	-	-
2001	526 (±3.04)	189 (±2.96)	227 (±1.86)
2002	874 (±5.31)	30 (±0.57)	-
2003	163 (±1.49)	-	548 (±6.88)
2004	-	780 (±7.64)	215 (±1.78)
2005	305 (±3.00)	-	51 (±0.57)
2006	-	-	47 (±0.96)
2007	-	-	114 (±1.29)
2008	6072 (±92.61)	47 (±0.82)	297 (±2.92)
2010	1669 (±18.46)	-	5140 (±84.81)
2011	-	1583 (±28.83)	5556 (±129.5)
2012	-	678 (±9.07)	1470 (±13.01)
2013	1723 (±15.03)	324 (±6.23)	3227 (±27.87)
2014	5304 (±51.78)	1192 (±8.92)	6058 (±46.37)

### Maturity and sex ratio

**Fig 7:**
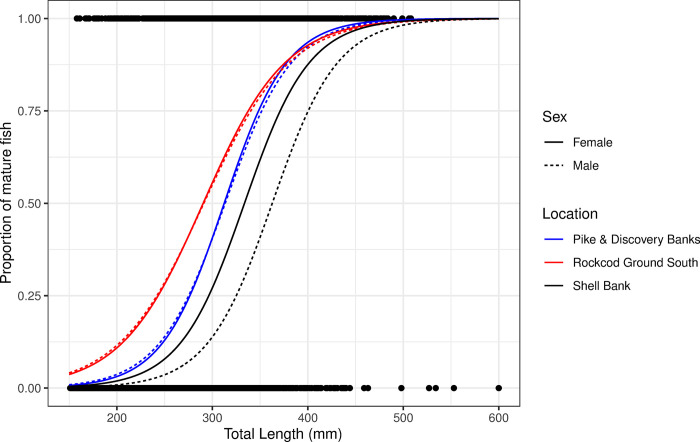
Maturity rates by length for male and female grey rockcod (*Lepidonotothen squamifrons*) caught in three locations within the vicinity of Heard Island and McDonald Islands.

No significant difference was detected between males and females from Rockcod Ground South (DF = 2, Deviance = 0.19, *p*>0.05) and Pike & Discovery Banks (DF = 2, Deviance = 0.15, *p*>0.05). The estimated length at 50% maturity was 290 mm and 313 mm, respectively for those two regions. In contrast, grey rockcod caught at Shell Bank showed significant differences in size at maturity between males and females (DF = 2, Deviance = 56.1, *p*<0.001), with females maturing at a smaller size than males (334 mm and 363 mm, respectively; Fig 7).

The sex ratio (males:females) at each location were close to 50:50 for Shell Bank (49.5:50.5) and Pike & Discovery Banks (49.2:50.8). Rockcod Ground South showed a more marked difference from the expected with more females than males present (44.5:55.5). ANOVA showed strong evidence of difference between locations (DF = 2, Deviance = 15.8, *p*<0.001), with pairwise comparison showing that Rockcod Ground South being different from the other two locations.

### Age estimation

**Fig 8:**
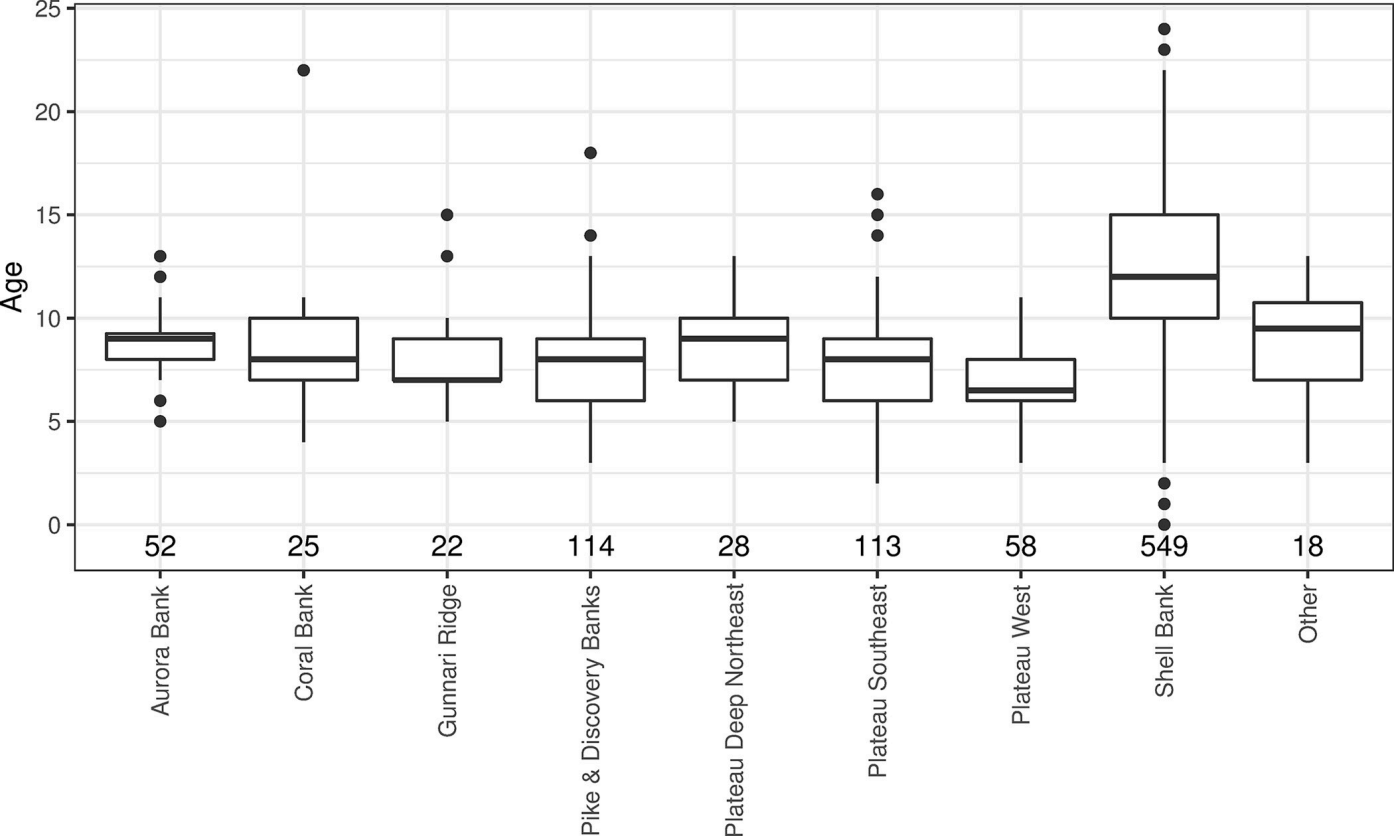
Box and whisker plots of age class distributions obtained from otoliths of grey rockcod (*Lepidonotothen squamifrons*) across locations within the vicinity of Heard Island and McDonald Islands. Note, locations with less than 10 samples have been grouped as ‘other’.

A total of 1010 otoliths were processed with 979 of these considered suitable quality for age estimation purposes. Grey rockcod age estimates ranged between 0+ to 24+ years (Fig 8). Individual readers differed on 66 of the 200 test otoliths, although differences were small with 20 of these being aged 1 year older by the experienced reader (BF) and 46 otoliths being aged one year younger. A Kolmogorov-Smirnov test between readers that showed no significant difference in the distribution of ages between readers (*p*>0.05). In addition to this the IAPE and CV were calculated at 1.84% and 2.61% respectively, as such, data from primary reader was used for all subsequent modelling.

### Growth curves and subpopulation comparisons

**Fig 9:**
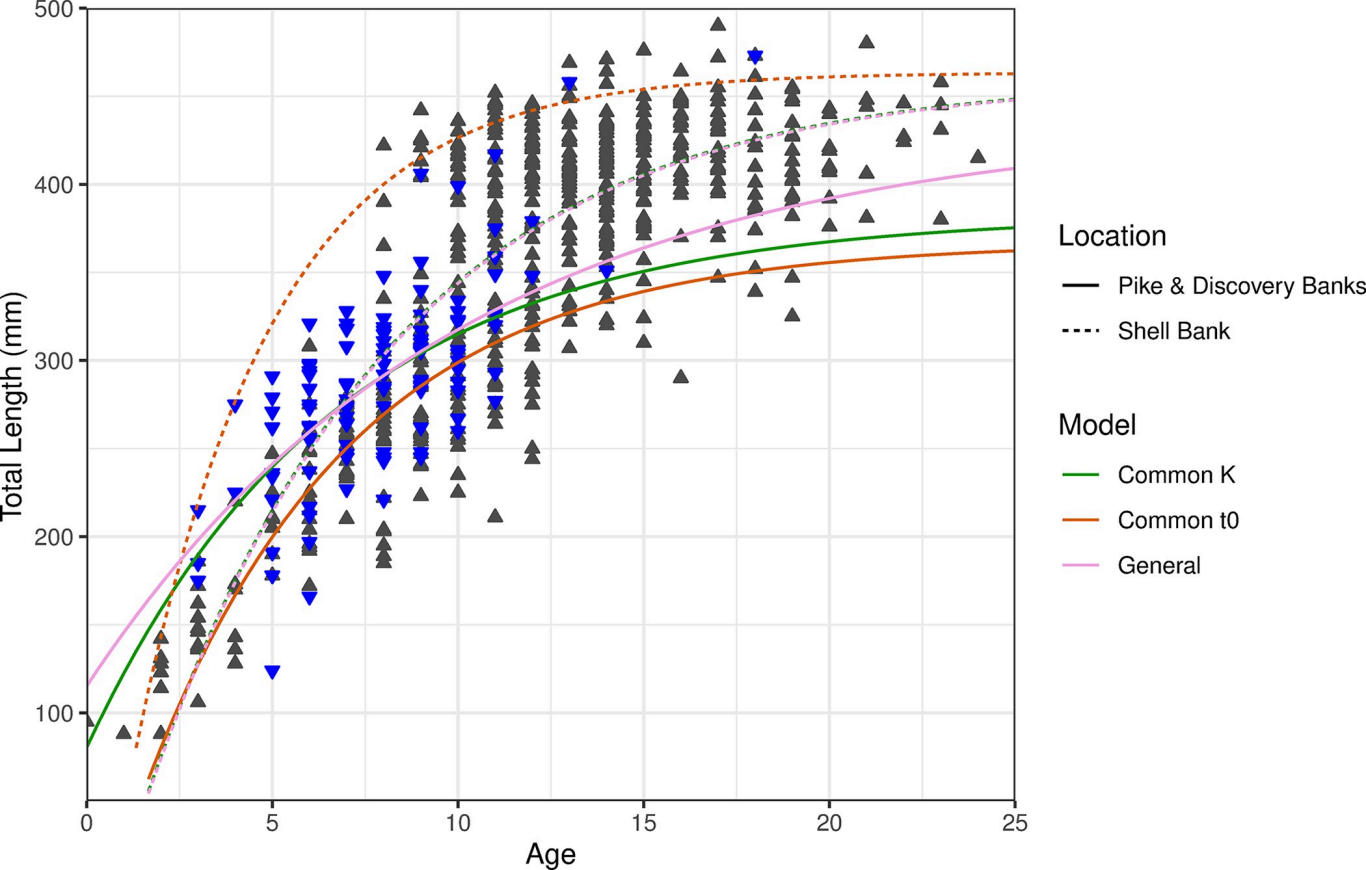
Varying parameter von Bertalanffy growth curves for grey rockcod (*Lepidonotothen squamifrons*) at Shell Bank (dashed lines and grey ▲ n = 549) and Pike & Discovery Banks (solid lines and blue ▼ n = 114) within the vicinity of Heard Island and McDonald Islands. Note: pink and green dashed lines sit on top of each other.

Differences in growth model parameters were identified between the two regions for which there was sufficient data. Grey rockcod at Shell Bank grow faster and attain a larger asymptotic length than those at Pike & Discovery Banks in the General and Common t_0_ models (Table 3, Fig 9). A von Bertalanffy model with a common *k* value and separate *L*_*∞*_ and *t*_*0*_ between regions was preferred, having lowest Akaike Information Criterion (AIC; Table 3) and the general model (no common parameters) was also plausible since it was within 2 AIC units of the optimal model. More broadly, the general model and those with one parameter in common were clearly preferred (11–20 AIC units) to models with two or all parameters in common.

**Table 3:** Comparison of von Bertalanffy model parameters of grey rockcod (*Lepidonotothen squamifrons*) growth curves for Shell Bank and Pike & Discovery Banks within the vicinity of Heard Island and McDonald Islands compared using Akaike Information Criterion (AIC).

Model	Shell Bank			Pike & Discovery Banks			Akaike Information Criterion		
	*K*	L_∞_	t_0_	*K*	L_∞_	t_0_	Parameters	AIC	δ AIC
Common values	0.13	473.10	0.14				3	6248.23	18.79
Separate *k* value	0.12	469.50	-2.22	0.13	469.50	-2.22	4	6246.12	16.68
Separate L_∞_ value	0.13	445.01	0.06	0.13	466.61	0.06	4	6243.54	14.10
Separate t_0_ value	0.13	473.18	0.04	0.13	473.18	0.17	4	6249.96	20.52
**Common *k* value**	**0.15**	**460.55**	**0.81**	**0.15**	**382.46**	**-1.57**	**5**	**6229.44**	0.00
Common t_0_ value	0.27	463.46	0.63	0.18	366.81	0.63	5	6232.42	2.98
General (all values separate)	0.15	460.09	0.83	0.10	435.37	-3.07	6	6231.22	1.78

Note: Lowest AIC is the optimal model (bold).

Investigating individual model effects within each region for common values of *k*, *t*_*0*_ or no common values (maximally parameterised (General) model) showed model fits for common *k* and maximally parameterised model within Shell Bank to be directly overlaid when plotted. Pike & Discovery Banks showed differing growth curves, intersecting at 6+ and *ca*. 250 mm for these two models. Models using a common *t*_*0*_ showed no overlap with other models from the same location, with Shell Bank having a higher *L*_*∞*_ than that of Pike & Discovery (Fig 9).

The biological significance of differences in growth models were also explored by comparing predicted length in the 8+ and 14+ age classes in order to visualise expected length differences between models. Pike & Discovery Banks showed differences between models occurring over an approximately 25 mm range for both age classes whereas large variation was seen between the common *t*_*0*_ model and the other models at Shell Bank which produced very similar expected lengths at age. In all three models fish at Shell Bank were expected to be bigger at both 8+ and 14+ than those at Pike & Discovery Banks (see S2 Fig).

## Discussion

### Population parameters derived from otoliths

This study is the first to use the increments in otoliths to estimate age and growth rates for grey rockcod revealing individuals aged between 0+ and 24+. Models estimating growth based on these age readings for Shell Bank and Pike & Discovery banks showed a model preference for a common *K* value with differences in *t*_*0*_ and *L*_*∞*._ This indicates that whilst the growth coefficient of both areas was the same the asymptotic length between the areas was quite different.

It was beyond the scope of this study to comprehensively validate the interpreted grey rockcod ages, or to compare data derived from scales as opposed to otoliths. However, this process of validation has been conducted for Patagonian toothfish (*D*. *eleginoides*) and marbled rockcod (*Notothenia rossii*), which have similar taxonomy, otolith morphology and habitat to grey rockcod [47–50]. In addition, the growth and age rates estimated in this study were consistent with those estimated for other demersal notothenioids (e.g. *Notothenia corriceps*, *N*. *rossii* and *D*. *eleginoides* [48,50–52]). Nonetheless, while further validation such as using tag mark recapture experiments, or tracking abundance cohorts would assist with confirming the growth rates estimated in this study would be beneficial, the low catch rates of the species in the longline fishery however seems to rule out any sort of mark recapture, including strontium marking. Any future work would thus rely heavily on future trawl surveys if an experiment of this nature were to be conducted. Given the likelihood that the increments viewed in otoliths for this species are formed annually these results increase the maximum age for this species within the Indian Ocean region compared to two previous studies on this species which used scale increments to estimate age and derive growth rates ranging between 0+– 19+ [23] and 1+– 15+ respectively [53].

There are several factors which may explain these differences. Comparative studies using otoliths and scales to estimate age have established that age estimates derived from scales have a tendency to underestimate of age due to the fact that scales can be shed and regrown during a fish’s life [42,54] and therefore the differences seen between our study and previous studies may be a result of the differing methodology. Age structure within an overexploited stock compared to that of an unexploited stock can be expected to show differences [1] As such it is unsurprising to see differences in ages between this study and the Duhamel and Ozouf-Costaz [23] study which occurred after the fishery was developed and concluded, however the sampling by Zaitsev [53] should have been a closer estimation of an unexploited population due to the timing of sample collection (1969–1986). This study has also shown that populations of the same species may have differing characteristics within a small spatial scale, as such it may be that grey rockcod populations within the vicinity of Heard Island and McDonald Islands may simply live longer than those in the region of Kerguelen Island.

### Population structure and connectivity

The HIMI area contains large sections of marine protected areas designed to protect biodiversity [55,56]. Substantial parts of Shell Bank and Pike & Discovery Banks strata, are within the HIMI Marine Reserve (83.82%, 75.68% respectively) where commercial fishing is prohibited, whereas Rockcod Ground South is open to commercial fishing. Coral and Aurora Banks are contained within the western most marine reserve and have had little sampling effort as a result of this. The spatial distribution of the grey rockcod catch indicated at least three distinct areas of consistently encountered grey rockcod within the HIMI EEZ. These areas are located on the tops of Pike & Discovery Banks, Rockcod Ground South and Shell Bank. They are generally separated by deeper regions with little or no evidence of demersal grey rockcod. All three of these locations are typically characterized by areas of harder rocky substrate, whist the areas between them are typically softer sediments [56].

On the northern section of the Kerguelen Plateau, Koubbi et al [57] reported ontogenic migration being a cause for grey rockcod to aggregate for spawning, this may be due to the northern part of the plateau having a larger amount of habitat compared to the more ‘bank like’ structure of HIMI; however, as our sampling showed all life stages and size classes present, it is unlikely the observed pattern is a result of ontogenic migration. The first report of geographically distinct sub-populations was made in the Atlantic sector by Gregory et al. [11] who noted the presence of ‘hot spots’ of grey rockcod to the east of Shag Rocks and the west of South Georgia in an otherwise patchy distribution.

In contrast with previous studies (e.g. [23,25,26]) which considered this species to be a single population spread over the Indian Ocean sector of the Southern Ocean, our study was the first to explore potential differences across locations within this area.

**Fig 10:**
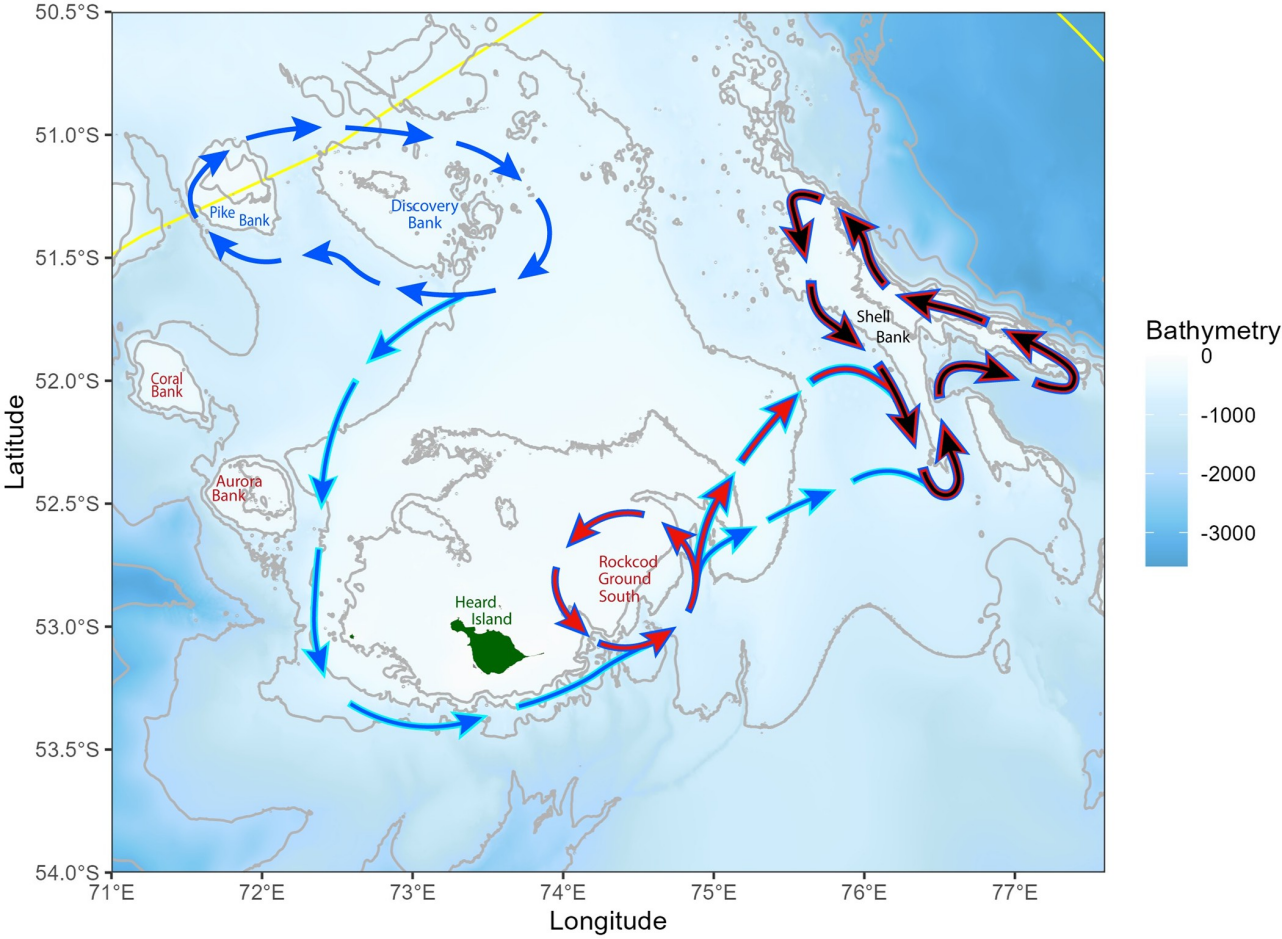
Potential connectivity pathways between three geographical populations of grey rockcod (*Lepidonotothen squamifrons*) within the vicinity of Heard Island and McDonald Islands (green). Scenarios indicate recruitment sources (blue and red arrows) and recruitment sinks (blue, red, black arrows at Shell Bank). Note light blue outlined arrows indicate pelagic recruits transported by oceanographic features adapted form Park et al., [60], bathymetry data obtained from Geoscience Australia [30].

This study revealed differences in estimated growth, size at maturity, sex ratio, body condition (weight at a given length) and population density among the three different areas. These findings support the hypothesis that each location is to some degree isolated from the others and as such supports the existence of geographically distinct demersal metapopulations, whilst not discounting the potential for larval mixing both from these areas but also outside sources (see Fig 10). The findings suggest that their contrasting biology is a response to local environmental conditions and/or metapopulation characteristics. It also highlights the plasticity of this species, which is consistent with other Sub-Antarctic species such as mackerel icefish (*Champsocephalus gunnari*), and may be a reason why the notothenoids are so successful in exploiting the new ecological space available in the Sub-Antarctic/ Antarctic that arose ~20 million years ago with the opening of Drake Passage [5].

This information, as well as incorporating other published studies on the biology of grey rockcod and the local geography of the region, allowed us for the first time to develop a hypothesis on contributing factors for the observed population structure of the grey rockcod within the HIMI area, and the implications of this hypothesis for vulnerability to over fishing and management.

The diet of grey rockcod consists primarily of salps and tunicates, and to a lesser extent amphipods and euphausids, much of which are derived from the mixed layer [11,58]. The best habitat for the grey rockcod would appear to be shallow waters (such as the top of banks) where productivity is likely to be high from nutrient upwellings possibly involving complex benthic habitats (such as sponge beds) for spawning and nesting ‘upstream’ from competitors [23,48,57,59]. Through benthic spawning, grey rockcod reduce the exposure of their eggs to predation compared to being amongst the plankton, as well as reducing the likelihood of being swept away by currents from suitable adult habitat [59]. This however, also means that dispersal between ‘hot spots’ or re-colonisation of suitable habitats that were previously over-exploited may be less likely than would occur if eggs were planktonic. Being benthic spawners also implies that historical unregulated demersal trawling may have inadvertently destroyed attractive spawning locations in conjunction with the over-exploitation of the species. The early life stages (post hatched larvae and fry) of the grey rockcod within the Kerguelen Islands as observed by Koubbi et al. [57] involve approximately 3–5 months within the water column. As such, primary recruitment of juveniles into any location is likely to be endogenous, with occasional downstream leaking to other locations (Fig 10), facilitated by local sea surface currents, such as those described by Park et al., [60] pushing larvae from one area to another.

These life cycle observations may explain the slow recovery of this species along with similar species such as marbled rockcod from overfishing in other locations [5]. Recovery may rely not only on a stock-recruitment relationship, but also on connectivity with other locations, and on food supply in suitable habitat patches. If a critical link in the chain of populations is over-exploited, the suitable spawning habitat destroyed, or supply of gelatinous zooplankton perturbed, then a recovery of a species such as grey rockcod could be delayed, or may never return to levels that supported unregulated fishing in the 1970’s, despite the absence of any fishing pressure for several generations [61].

It is clear that the metapopulation identified here would contribute to the vulnerability of a population to overexploitation if all three populations are considered to have the same parameters. We contend that such cryptic metapopulation structure may also be a contributing factor to the slow recovery of other Southern Ocean species, or those such as Orange Roughy (*Hoplosthetus atlanticus*) that may rely on widely separated habitat patches such as seamounts. What is not clear for the grey rockcod is if the meta-population structure is a result of fragmentation of a single wide spread pre-exploitation population as suggested by Kenchington [4] or the re-establishment of the structure that existed prior to fishing. Tagging, genetic studies and the investigation of relationships between recruitment, environmental conditions and spawning habitat quality would be useful in the further evaluation of this potential connectivity pathway (such as those described in [16,28,61,62]). In the meantime, we suggest that for the purposes of fisheries management, each metapopulation should be considered separately, as they appear to have their own independent dynamics.

## Conclusion

Grey rockcod was among the first fish species on the Kerguelen Plateau to be exploited by industrial fisheries [7,41]. It has not been targeted by fishing within the HIMI area since 1981 and very little was known about the population dynamics of this species within the Australian EEZ. Mapping of research survey catches indicated the occurrence of three distinct geographical sub-populations. Further investigation showed differences in their biological parameters such as body condition, sex ratio, maturity, population density, growth and observed age range. These results lead us to conclude that the HIMI EEZ likely contains at least three metapopulations of grey rockcod, and the differences in their metapopulation biology may have contributed to the slow recovery of this species despite very low levels of fishing mortality for several decades.

In addition to updating a range of biological parameters this study has also increased the estimated maximum age of the grey rockcod from 19 to 24 years. Future work on this species in this area should idea include genetics work, and a tagging study to explore connectivity between metapopulations. Additionally, the tagging study would aid in validation of the formation of annuli in otoliths.

## Supporting information

S1 Fig Transverse section of a *Lepidonotothen squamifrons* otolith, total length: 223 mm. Red dots indicate annual growth rings.
(TIF)

S2 Fig Length comparison of *Lepidonotothen squamifrons* for the 8+ and 14+ age classes under varying parameter von Bertalanffy models for Pike & Discovery Banks (blue) and Shell Bank (black) within the vicinity of Heard Island and McDonald Islands. (TIF)

S1 Table Details of research surveys carried out within the vicinity of Heard Island & McDonald Islands between 1990 and 2014. Vessels names are Aurora Australis (AA), Austral Leader (AL) and Southern Champion (SC).
(DOCX)

S2 Table Estimates of the length-weight parameters of *Lepidonotothen squamifrons* using data collected from three locations within the vicinity of Heard Island and McDonald Islands during research surveys, and combined years. (DOCX)
