# Supplementary figures and images for: Population dynamics and spatial structure of the grey rockcod (Lepidonotothen squamifrons) in the vicinity of Heard Island and the McDonald Islands
Source: PLoS One. 2024 May 14;19(5):e0298754. doi: 10.1371/journal.pone.0298754 (PMC11093291; doi:10.1371/journal.pone.0298754)

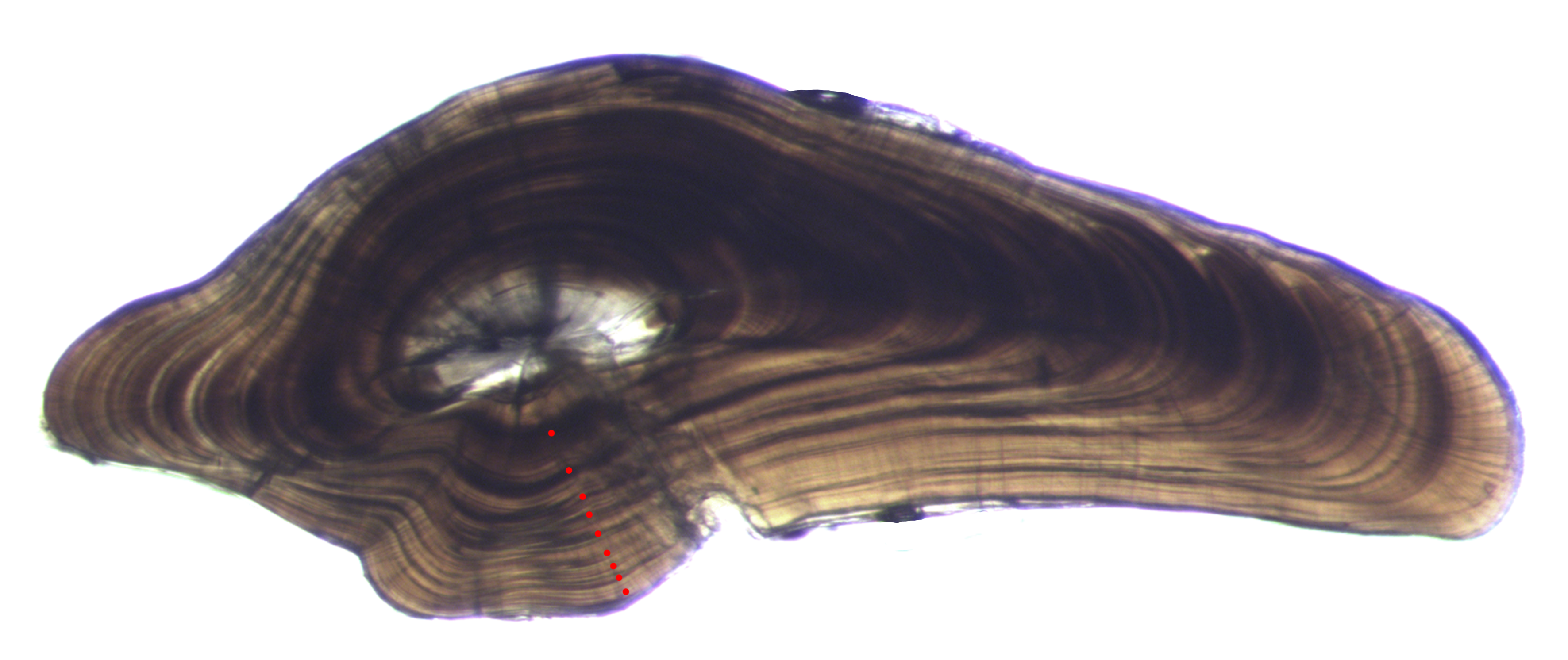

Supplement: S1 Fig — Red dots indicate annual growth rings. (TIF) [file pone.0298754.s001.tif]

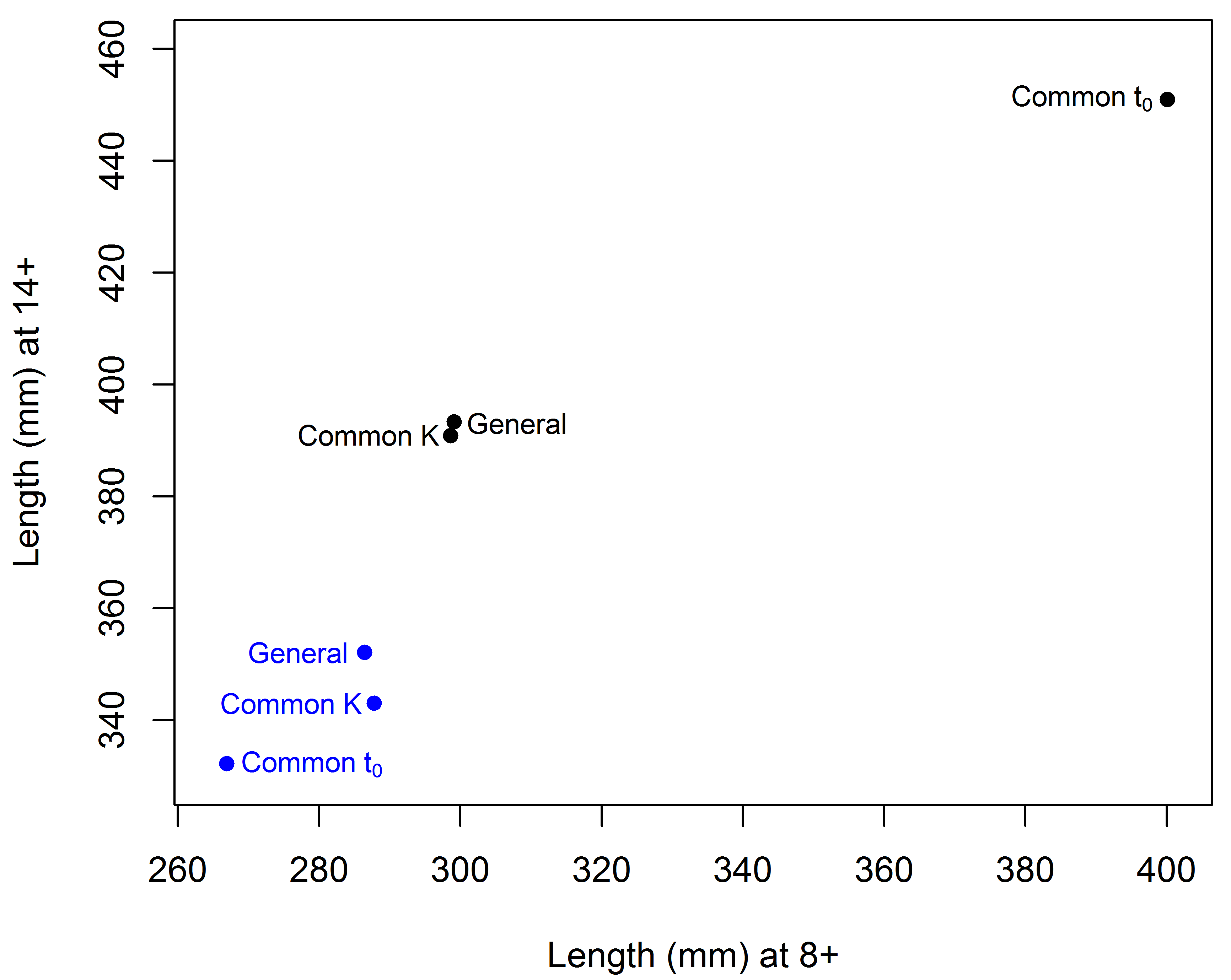

Supplement: S2 Fig — (TIF) [file pone.0298754.s002.tif]
