# Supplementary material for: Population dynamics and spatial structure of the grey rockcod (Lepidonotothen squamifrons) in the vicinity of Heard Island and the McDonald Islands
Source: PLoS One. 2024 May 14;19(5):e0298754. doi: 10.1371/journal.pone.0298754 (PMC11093291; doi:10.1371/journal.pone.0298754)
Supplement: S1 Table — Vessels names are Aurora Australis (AA), Austral Leader (AL) and Southern Champion (SC). (DOCX) [file pone.0298754.s003.docx]

**S1 Table. Details of research surveys carried out within the vicinity of Heard Island & McDonald Islands between 1990 and 2014.** Vessels names are Aurora Australis (AA), Austral Leader (AL) and Southern Champion (SC)

| Year | Stations | Vessel |
| --- | --- | --- |
| 1990 | 79 | AA |
| 1992 | 68 | AA |
| 1993 | 66 | AA |
| 1997 | 40 | AL |
| 1998 | 40 | AL |
| 1999 | 150 | SC |
| 2000 | 105 | SC |
| 2001 | 120 | SC |
| 2002 | 132 | SC |
| 2003 | 112 | SC |
| 2004 | 145 | SC |
| 2005 | 158 | SC |
| 2006 | 158 | SC |
| 2007 | 159 | SC |
| 2008 | 159 | SC |
| 2009 | 162 | SC |
| 2010 | 134 | SC |
| 2010 | 158 | SC |
| 2011 | 158 | SC |
| 2012 | 174 | SC |
| 2013 | 158 | SC |
| 2014 | 164 | SC |
