# Supplementary material for: Population dynamics and spatial structure of the grey rockcod (Lepidonotothen squamifrons) in the vicinity of Heard Island and the McDonald Islands
Source: PLoS One. 2024 May 14;19(5):e0298754. doi: 10.1371/journal.pone.0298754 (PMC11093291; doi:10.1371/journal.pone.0298754)
Supplement: S2 Table — (DOCX) [file pone.0298754.s004.docx]

**S2 Table. Estimates of the length-weight parameters of *Lepidonotothen squamifrons* using data collected from three locations within the vicinity of Heard Island and McDonald Islands during research surveys, and combined years.**

|  | Pike & Discovery Banks | | | Shell Bank | | | Rockcod Ground South | | |
| --- | --- | --- | --- | --- | --- | --- | --- | --- | --- |
| Year | n | *a* | *b* | n | *a* | *b* | n | *a* | *b* |
| 1990 | 47 | 1.13E-07 | 3.81 | 254 | 1.44E-05 | 2.98 |  |  |  |
| 1992 | 18 | 8.09E-08 | 3.86 | 707 | 6.64E-06 | 3.11 |  |  |  |
| 2000 |  |  |  | 3202 | 1.60E-06 | 3.34 |  |  |  |
| 2001 | 116 | 1.61E-06 | 3.37 | 275 | 5.02E-06 | 3.15 | 177 | 1.80E-05 | 2.92 |
| 2002 |  |  |  | 424 | 3.27E-06 | 3.22 |  |  |  |
| 2003 |  |  |  | 131 | 3.53E-06 | 3.21 | 409 | 4.65E-07 | 3.57 |
| 2004 | 126 | 1.64E-06 | 3.35 |  |  |  | 254 | 9.71E-07 | 3.43 |
| 2005 |  |  |  | 155 | 6.06E-07 | 3.51 | 54 | 1.49E-06 | 3.34 |
| 2006 |  |  |  | 31 | 7.48E-07 | 3.49 | 56 | 8.21E-07 | 3.46 |
| 2007 | 24 | 4.10E-08 | 4.01 | 259 | 7.98E-07 | 3.47 | 47 | 7.29E-07 | 3.48 |
| 2008 | 23 | 1.52E-06 | 3.36 | 213 | 1.50E-06 | 3.36 | 188 | 3.30E-07 | 3.63 |
| 2010 | 394 | 4.10E-08 | 4.01 | 169 | 3.16E-07 | 3.63 | 1117 | 8.58E-07 | 3.45 |
| 2011 | 38 | 9.72E-07 | 3.44 | 111 | 6.98E-06 | 3.09 | 133 | 1.56E-07 | 3.75 |
| 2012 | 161 | 8.93E-07 | 3.45 | 152 | 1.12E-06 | 3.41 | 1809 | 1.32E-06 | 3.38 |
| 2013 | 44 | 1.15E-06 | 3.40 | 181 | 3.43E-06 | 3.21 | 810 | 3.86E-07 | 3.59 |
| 2014 | 260 | 6.21E-07 | 3.52 | 849 | 2.15E-06 | 3.29 | 635 | 3.50E-07 | 3.60 |
| Combined | 1251 | 5.29E-07 | 3.55 | 7113 | 2.75E-06 | 3.25 | 5765 | 1.80E-06 | 3.32 |
